# COVID-19 in renal transplant recipients and general population: a comparative study of clinical, laboratory, and radiological features, severity, and outcome

**DOI:** 10.1186/s12985-021-01713-x

**Published:** 2021-12-07

**Authors:** Ali Monfared, Leila Akhondzadeh, Mahsa Mousazadeh, Atefeh Jafari, Masoud Khosravi, Mohammadkazem Lebadi, Pegah Aghajanzadeh, Yalda Haghdar-Saheli, Ali Movassaghi, Elham Ramezanzadeh, Farzaneh Shobeirian, Ehsan Kazemnezhad, Samaneh Esmaeili

**Affiliations:** 1 Urology Research Center, School of Medicine, Razi Hospital, Guilan University of Medical Sciences, Rasht, Iran; 2 School of Medicine, Guilan University of Medical Sciences, Rasht, Iran; 3 Department of Clinical Pharmacy, School of Pharmacy, Guilan University of Medical Sciences, Rasht, Iran; 4 Department of Radiology, Guilan University of Medical Sciences, Rasht, Iran

**Keywords:** COVID-19, Kidney transplantation, Transplant recipients, Immunosuppression

## Abstract

**Introduction:**

Coronavirus disease 2019 (COVID-19), a novel disease caused by severe acute respiratory syndrome coronavirus 2 (SARS-CoV-2), has led to millions of deaths worldwide. Kidney transplant recipients (KTRs) are a fragile population due to their immunosuppressed status. However, there are limited studies available comparing this population with the general population regarding clinical symptoms, and laboratory and imaging features as well as disease severity and clinical outcomes.

**Methods:**

A total of 24 KTRs and 40 patients from the general population (control group) were enrolled after applying exclusion criteria. Clinical symptoms, laboratory values, and lung involvement patterns in high-resolution computed tomography (HRCT) were compared between KTRs with COVID-19 and their counterparts from the general population. Moreover, the category of disease severity and adverse outcomes such as intensive care unit (ICU) admission, mechanical ventilation (MV), and mortality rate were also compared between these two groups.

**Results:**

Hypertension was significantly higher among KTRs. Dyspnea was significantly more among the control group (*P* = 0.045). There was no significant difference in the rest of clinical symptoms (*P* > 0.05). There was no significant difference in CT features as well, except pleural effusion, which was more prevalent in the control group. A lower absolute lymphocytic count (ALC) and platelet count were observed in KTRs. Renal transplant recipients (RTRs) had a higher elevation in creatinine level than their counterparts. The ICU admission, MV, duration of hospital stay, and mortality as adverse outcomes were not significantly different between the KTR and control groups.

**Conclusion:**

In conclusion, there was no significant difference in the severity and risk of adverse outcomes, including MV, ICU admission, and mortality between KTRs under chronic immunosuppression and the control group.

## Introduction

Coronavirus disease 2019 (COVID-19) infection is caused by severe acute respiratory syndrome coronavirus 2 (SARS-CoV-2). It was first identified when a cluster of cases of pneumonia were reported in December 2019 in Wuhan province, China. Since then, it had spread rapidly throughout the world, and in January 2020, the WHO declared this novel coronavirus (2019-nCoV) a Public Health Emergency of International Concern (PHEIC) [1].

At the time of data collection in February 2020, there were 82 623 infected cases and 2858 deaths worldwide. By June, 2020, the number sharply increased with more than 6.2 million confirmed cases and 370 000 deaths worldwide [2, 3].

The disease initially presents as an acute respiratory infection with alveolar and interstitial pneumonia; however, it can affect other organs such as the kidney, heart, gastrointestinal tract, hematological system, central nervous system, and musculoskeletal system [4].

The SARS-CoV-2 infection induces a wide spectrum of clinical courses comprising asymptomatic infection, mild respiratory symptoms, severe respiratory infection, and multiorgan damage. Approximately 80% of patients show a mild clinical status; however, elderly (≥ 65 years) and patients with comorbid conditions and immunosuppression are at increased risk of rapid progress to severe and critical illness requiring intensive care unit (ICU) admission [5, 6]. Viral infection in patients who have undergone organ transplantation poses a significant risk of morbidity and death [3].

With more research going into this disease, our knowledge about its features and management in high-risk groups such as renal transplant recipients (RTRs) is also increasing [7].

There are reports of mortality rate in the range 20%–28% among RTRs and 1%–5% among non-RTRs with COVID-19 infection [8].

Organ transplant recipients are generally found to be more susceptible to severe features and complications of viral pneumonia owing to their immunosuppressed status [9, 10]. Besides, concurrent comorbidities in these patients such as diabetes mellitus, hypertension (HTN), hyperlipidemia, and cardiovascular and cerebrovascular disease escalate this possibility [6, 11].

On the other hand, an immense proinflammatory state, which is responsible for organ dysfunction and acute respiratory distress syndrome (ARDS) during severe COVID-19 infection, is potentially mitigated by the immunosuppressant regimes prescribed to RTRs as a maintenance therapy preventing allograft rejection [7, 10, 12].

However, it is still debated whether the use of immunosuppressive drugs in KTRs could deteriorate the situation into adverse outcomes or protect this population against severe features of SARS-CoV-2 infection.

Comparison of the clinical, laboratory, and radiological data for RTRs with COVID-19 with those obtained for their counterparts in the general population has scarcely been taken into consideration so far.

A limited number of studies have compared the clinical course and outcomes of COVID-19 infection in KTRs versus non-KTRs [13–15].

In a recent study, it was found that hemodialysis, chronic kidney disease, and renal transplant patients had a higher mortality rate than those without any underlying kidney disease [13], while another observational study conducted in the United States revealed a greater possibility of ICU admission in KTRs compared to the general population without a difference in risk of mortality [14]. In another study carried out at the onset of the outbreak, clinical, laboratory, and imaging features of 10 renal transplant patients were found similar to those of 10 patients in the control group having severe pneumonia [15].

Nevertheless, to the best of our knowledge, a comparative study of both clinical and paraclinical features encompassing clinical course, outcomes as well as radiological characteristics between RTRs and a control group with a larger sample size is still lacking. Hence, we conducted this case–control study to compare not only clinical symptoms, disease severity, and outcomes but also lung imaging features between RTRs and a control group of non-RTRs with COVID-19 hospitalized in our medical center.

## Materials and methods

### Study setting

The present study was performed at Razi Hospital, an educational, therapeutic, and research center where a good number of kidney transplant surgeries are performed annually.

### Study population

A case group of 24 KTRs and a control group of 40 non-KTRs—with a COVID-19 diagnosis—hospitalized from February 2020 through June 2020 were included.

COVID-19 was confirmed in the study population based on a positive reverse transcription-polymerase chain reaction (RT-PCR) assay for SARS-CoV-2 of the nasopharyngeal swab specimens and/or typical pulmonary lesions on high-resolution computed tomography (HRCT) [16].

In the control group, patients under active immunosuppressive therapy, chemotherapy, or who were known cases of malignancy or chronic kidney disease were excluded. Patients were risk stratified based on the severity of infection as mild, moderate, severe, and critically ill on presentation to the hospital, using the WHO-China Joint Mission Classification [17].

- Mild: no findings of pneumonia on HRCT and presence of mild clinical symptoms.
- Moderate disease: HRCT manifestations compatible with viral pneumonia and presence of respiratory symptoms and fever.
- Severe: patients with any of the following evidence: respiratory rate ≥ 30 beats per minute or respiratory distress; O_2_ saturation ≤ 93% in a resting state; or oxygen concentration (FiO_2_) or partial pressure of arterial blood oxygen (PaO_2_) ≤ 300 mmHg.
- Critical: having any of the following evidence: occurrence of respiratory failure and requiring mechanical ventilation (MV), shock, and other organ failure requiring ICU admission and monitoring.

### Study design

This was a case–control study comparing demographic data, clinical symptoms, paraclinical findings, and composite outcomes between KTRs and non-KTRs admitted due to COVID-19.

The study protocol was approved by the ethics committee of the Guilan University of Medical Sciences (approval number: IR.GUMS.REC.1399.251).

Due to the critical situation in our center which has been engendered by the rapid and sudden spread of COVID-19 infection, not all patients were consented initially at the time of admission; however, those who hadn’t been consented during the admission period, were consented in follow up visits. (Reviewer 2- comment 2).

### Immunosuppression management and antiviral therapy

Treatment plans, including immunosuppressive management and antiviral therapy, were based on the local protocol in our country published by the Ministry of Health. The general management strategy was to discontinue the overall dose of antimetabolites and reduce chronic renal failures (CNIs).

### Serum laboratory markers and RT-PCR

Laboratory examinations were performed on admission as well as during the admission period, including baseline C-reactive protein (CRP) level, erythrocyte sedimentation rate (ESR), creatinine, transaminases, serum electrolytes, cell blood counts, and differentials. The peak values were recorded over the admission period. Acute kidney injury (AKI) was identified according to the criteria from the Kidney Disease Improving Outcomes (KDIGO) [18].

RT-PCR test was performed using Novel Corona Virus (2019Cov) Nucleic Acid Diagnostic Kit. The manufacturer was Sansure Biotech. (Reviewer 2- comment 4).

### Imaging features

All patients underwent HRCT of the lungs on admission, due to its high sensitivity to SARS-CoV-2 infection [19]. All HRCT findings were interpreted by a board-certified radiologist. HRCT findings were deemed suspicious for COVID-19 pneumonia if typical patterns of basal, bilateral, and peripheral predominant consolidation, and ground-glass opacity (GGO) or both, often with a reversed halo sign, were detected [16]. Pure GGO, crazy-paving, mixed GGO and consolidation, pure consolidation, reversed halo, linear opacities, traction bronchiectasis, mediastinal lymphadenopathies, and pleural and pericardial effusion were all documented as patterns of lung involvement. The location of the opacities was considered as peripheral if it was in the outer one-third of the lungs; otherwise, it was considered as central.

Lung involvement was measured semi-quantitatively using the total CT score. Each involved lobe was scored based on the following: 0 = 0% involvement; 1 = less than 5% involvement; 2 = 5%–25% involvement; 3 = 26%–49% involvement; 4 = 50%–75% involvement; and 5 =  > 75% involvement. There was a score of 0–5 for each lobe, with a total CT score ranging from 0 to 25 [20].

### Data collection

Required data, including demographics, clinical symptoms, laboratory parameters, HRCT findings, medications and changes, type of ventilation, ICU admission, length of admission, and discharge status, were all documented in checklists.

### Outcome measures

ICU admission, MV requirement, and in-hospital mortality were considered as adverse outcomes.

### Statistical analysis

Based on the normality, data were reported as mean ± SD or median and interquartile range (IQR).

For comparing continuous variables, Mann–Whitney U test or *t*-test was done, along with conducting Chi-squared or Fisher’s exact *t*-test for categorical data, as found appropriate.

Multivariate logistic regression analysis was performed to determine the association between study population status and outcomes of interest.

All statistical analyses were performed using SPSS (IBM SPSS Statistics 26). *P* ≤ 0.05 was considered statistically significant. (Reviewer 2- comment 7).

## Results

### Admission characteristics

In this study, after applying the exclusion criteria, 24 RTRs and 40 non-RTRs as the control-matched group, infected with COVID-19, were included.

As shown in Table 1, the mean and median age and the sex proportion were roughly matched and thus were not significantly different between the two groups. (*P* = 0.491 and *P* = 0.431, respectively).

**Table 1:** Demographic and past medical history and drugs in RTRs and nonRTRs

		Group			*P*
		Transplant Pts	Non-transplant Pts	Total	
		Count (N %)	Count (N %)	Count (N %)	
Age group	Low—50 yrs	11 (45.8)	16 (40.0)	27 (42.2)	0.491
	50—59 yrs	4 (16.7)	12 (30.0)	16 (25.0)	
	60—high yrs	9 (37.5)	12 (30.0)	21 (32.8)	
Age	52.25 ± 12.88 (29.00 – 76.00)	51.58 ± 11.32 (32.0 – 72.0)	51.83 ± 11.83 (29.0 – 76.0)		
Sex	Male	17 (70.8)	24 (60.0)	41 (64.1)	0.431
	Female	7 (29.2)	16 (40.0)	23 (35.9)	
HTN		20 (87.0)	16 (41.0)	36 (58.1)	< 0.001
Diabetes		11 (47.8)	12 (30.8)	23 (37.1)	0.179
CVA		1 (4.3)	1 (2.6)	2 (3.2)	0.608
IHD or HF		4 (17.4)	8 (20.5)	12 (19.4)	0.520
DVT		1 (4.3)	0 (0.0)	1 (1.6)	0.371
PTE		0 (0.0)	1 (2.6)	1 (1.6)	0.621
COPD		1 (4.3)	7 (17.9)	8 (12.9)	0.123
Cellcept		24 (12.9)		24 (100.0)	-
Cyclosporine		13 (56.5)		13 (56.5)	
Tacrolimus		9 (40.9)		9 (40.9)	
Sirolimus		1 (4.3)		1 (4.3)	
Corticosteroid		23 (95.8)	4 (10.0)	27 (42.2)	0.001
ARB or ACEi		6 (25.0)	13 (34.2)	22 (36.7)	0.444
Atorvastatin		20 (83.3)	13 (34.2)	31 (50.8)	0.001
Warfarin		1 (4.3)	2 (5.3)	3 (4.9)	0.684
HCQ		23 (95.8)	40 (100.0)	63 (98.4)	0.375
Kaletra		17 (70.8)	30 (75.0)	47 (73.4)	0.715
Tamiflu		16 (66.7)	21 (52.5)	37 (57.8)	0.267
Azithromycin		2 (8.3)	8 (20.0)	10 (15.6)	0.189
IVIG		4 (16.7)	0 (0.0)	4 (6.3)	0.017
Gancyclovir		1 (4.2)	0 (0.0)	1 (1.6)	0.375
Ribavirin		2 (8.3)	0 (0.0)	2 (3.1)	0.137

*Mean ± SD (Min—MAX)

HTN was identified as the most common comorbidity in both groups, which had a significantly higher prevalence in RTRs (87%) compared to the control group (41%) (*P* < 0.001). Apart from HTN, diabetes and cardiovascular disease were the other most frequent comorbidities in both groups.

Regarding clinical presentations, fever (73.9%) and cough (73.9%) were the most common clinical presentations in RTRs while dyspnea (90%) and cough (85%) were the most common symptoms in the control group. (Reviewer 2-comment 8).

Dyspnea was identified as the only clinical symptom that was present significantly more in the control group than in RTRs (90% versus 69.6%, respectively) (*P* = 0.045). (Table 2).

**Table 2:** Clinical and imaging finding in RTRs and nRTRs

	Group		*P*
	Transplant Pts	Non-transplant Pts	
	Count (n%)	Count (n%)	
Chills	11 (47.8)	13 (32.5)	0.228
NauseaVomiting	5 (21.7)	13 (32.5)	0.363
Diarrhea	5 (21.7)	12 (30.0)	0.477
Hemoptysis	1 (4.3)	0 (0.0)	0.189
Myalgia	6 (26.1)	15 (37.5)	0.355
headache	7 (30.4)	9 (22.5)	0.486
Anosmia	5 (21.7)	3 (9.4)	0.185
Fever	17 (73.9)	28 (70.0)	0.741
Cough	17 (73.9)	34 (85.0)	0.281
Dyspnea	16 (69.6)	36 (90.0)	0.045
Total GGO	22 (95.7)	38 (95.0)	0.701
Only GGO	2 (8.7)	6 (15)	0.381
Total consolidation	19 (82.6)	34 (85.0)	0.534
GGO + crazy paving	2 (8.7)	0 (0)	0.130
Only consolidation	1 (4.3)	2 (5)	0.701
GGO + consolidation	11 (47.8)	15 (37.5)	0.423
GGO + crazy paving + consolidation	7 (30.4)	17 (42.5)	0.342
Crazy paving	9 (39.1)	17 (42.5)	0.794
Multifocal bilateral	22 (95.7)	36 (97.3)	0.624
Multifocal unilateral	1 (4.3)	1 (2.7)	0.624
Unifocal	0 (0.0)	0 (0.0)	-
Peripheral	16 (100.0)	36 (97.3)	0.698
Central	6 (37.5)	10 (27.0)	0.327
Reversedhalo	9 (39.1)	15 (44.1)	0.708
Linear opacities	9 (39.1)	17 (50.0)	0.419
Bronchiectasis	5 (21.7)	10 (29.4)	0.519
Pleural effusion	0 (0.0)	8 (23.5)	0.013
Pericardial effusion	2 (9.1)	4 (11.8)	0.560
Number of involved lobes	4.65 ± 0.70 (5.00 (5.00–5.00))	4.32 ± 1.04 (5.00 (4.00–5.00))	0.318
Total CT score	12.40 ± 5.87 (11.00 (8.00–18.00))	11.59 ± 6.09 (10.50 (7.00–17.00))	0.576

### Medication

CellCept, cyclosporine, tacrolimus, sirolimus, and corticosteroids were immunosuppressive agents taken by 24 (100%), 13 (56.5%), 9 (40.9%), 1 (4.3%), and 23 (95.8%) RTRs, respectively.

Dose adjustment of immunosuppressant agents was applied in all 24 KTRs. CellCept was withdrawn later. Corticosteroid was continued with a stress dose in 23 KTRs. Calcineurin inhibitors were either reduced or discontinued in patients with a severe clinical status. Serum tacrolimus level was monitored in order to minimize the pharmacokinetic interaction with antiviral drugs (i.e. lopinavir/ritonavir) administered simultaneously.

A total of 18 (78.3%) RTRs and 13 (34.2%) non-RTRs were on statins prior to admission (*P* = 0.001).

Intravenous immunoglobulin (IVIG) administration was done in 4 (16.7%) RTRs, of which 1 patient eventually expired; however, none of the patients in the control group were prescribed IVIG (P = 0.017). (Table 1).

### Imaging

HRCTs of lung involvement are classified as shown in Table 2.

Patchy GGO and consolidation (with or without other findings) were the most common HRCT findings detected in both groups with no significant intergroup difference (*P* > 0.05).

Regarding lesion types, GGOs accompanied by consolidation and crazy-paving were a frequent finding in both groups, followed by reversed halo sign and crazy-paving; however, there was no significant difference between the groups (*P* > 0.05).

Consolidation alone appeared in a limited number of patients: 1 RTR (4.3%) and 2 in the control group (5%) (*P* = 0.701).

**Fig. 1:**
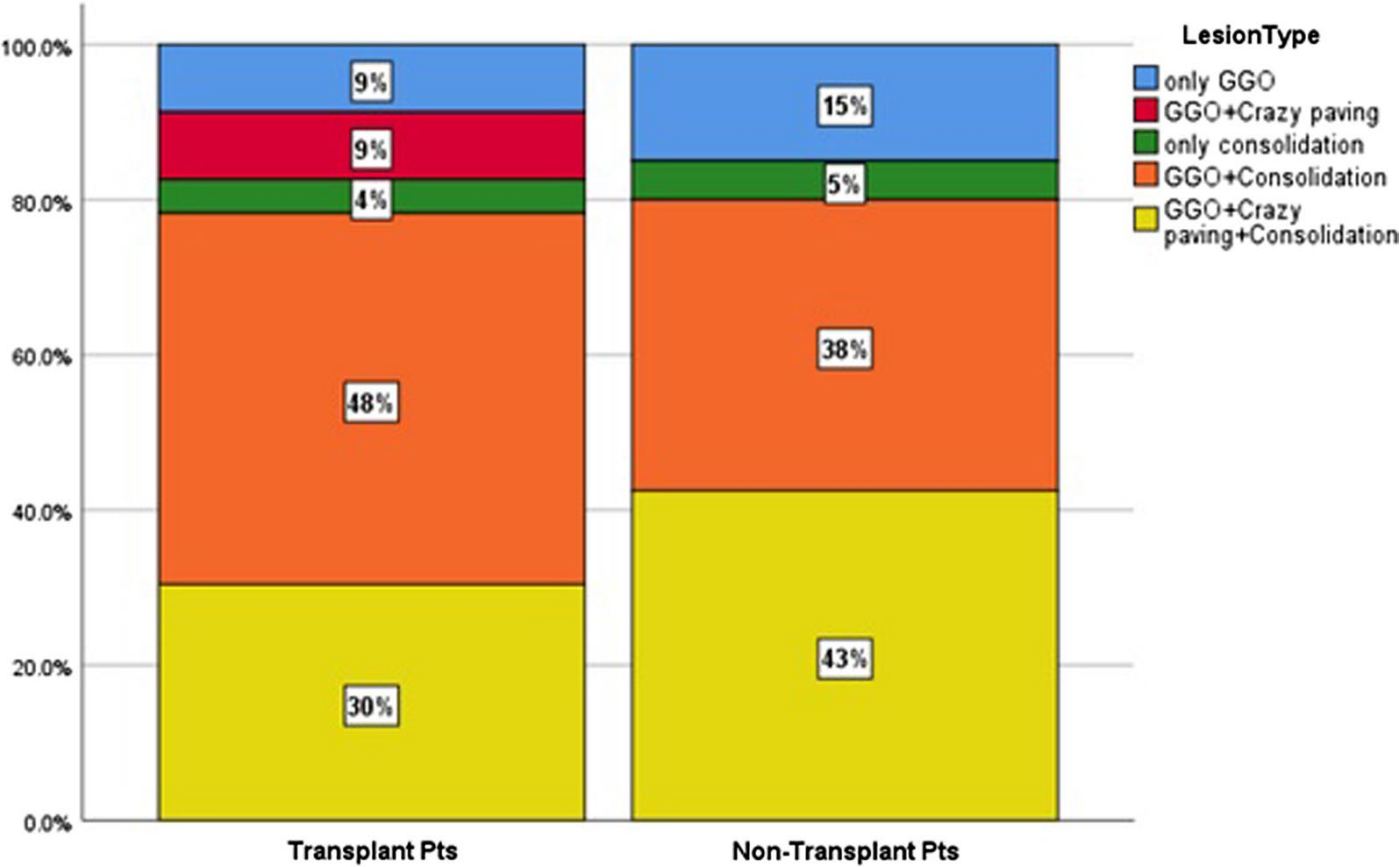
Radiologic findings in 2 groups

In the majority of patients in each group, lesions were found to be multifocal and distributed bilaterally, as well as more in the peripheral area than in the central, with no significant difference in the distribution of lesions intergroup (*P* > 0.05). (Fig. 1).

All HRCT manifestations were almost similar between RTRs and non-RTRs (*P* > 0.05), except pleural effusion, which was the only feature that appeared significantly more in the control group than in RTRs (8 [23.5%] versus 0%, respectively) (*P* = 0.013). (Reviewer 2- comment 6).

Total CT score as well as the number of involved lobes was not statistically different between the two groups (*P* = 0.576).

### Laboratory parameters

Laboratory parameters are shown in Table 3.

**Table 3:** Laboratory finding in RTRs and nonRTRs

		Group		*P*
		Transplant Pts	Non-transplant Pts	
		Count (N %)	Count (N %)	
CRP	Negative	4 (19.0)	10 (30.3)	0.252
	1 +	5 (23.8)	11 (33.3)	
	2 +	1 (4.8)	1 (3.0)	
	3 +	11 (52.4)	11 (33.3)	
Troponin	Negative	5 (100.0)	14 (93.3)	0.750
	Positive	0 (0.0)	1 (6.7)	
PCR	Negative	4 (16.7)	7 (17.5)	0.965
	Positive	6 (25.0)	11 (27.5)	
	Not checked	14 (58.3)	22 (55)	
WBC		7537.50 ± 5673.65, (6050 (4150–7050.00))	9772.50 ± 4719.95, 8400(6600 -1210)	0.094
Neutrophil Count		81.07 ± 5.55 (82.00(80.00–86.00))	78.70 ± 10.10 (80.00 (70.00–86.00))	0.334
Lymphocyte Count		12.87 ± 6.02 (11.00 (10.00–16.00))	18.00 ± 9.75 (17.00 (10.00–25.00))	0.072
ALC		820.93 ± 432.54, (795.00 (392.00–1134))	1794.89 ± 1376.59, (1417.00 (860.00–2057.00))	0.002
Hemoglubin		11.28 ± 2.59, (10.85 (9.00–12.85))	12.30 ± 1.79 (12.10 (11.00–13.40))	0.099
Platelets		165.13 ± 81.95, (133.00 (114.00–194.00))	269.10 ± 141.25 (246.50 (168.00–302.50))	0.002
ESR		63.70 ± 37.12 (77.00 (26.00–100.00))	67.95 ± 30.28 (66.50 (51.50–95.50))	0.739
Max Cratinine		2.59 ± 1.70 (1.75 (1.30–3.65))	1.10 ± 0.46 (1.00 (0.90–1.20))	0.001
Max LDH		846.78 ± 481.98 (621.50 (550 -1106))	807.27 ± 480.63 (683.00 (506.00–938.00))	0.790
CPK		362.38 ± 390.89 (263.00 (106.00–350.00))	428.93 ± 1235.04 (130.00 (74.00–230.00))	0.851
Na		131.50 ± 6.52 (131.00 (126.50–135.00))	134.92 ± 4.87 (135.00 (131.00–137.00))	0.20
K		4.21 ± 0.37 (4.30 (4.00–4.45))	4.35 ± 0.61 (4.35 (3.85–4.70))	0.254
Ca		8.16 ± 0.78 (8.20 (7.50–8.70))	8.38 ± 0.38 (8.50 (8.15–8.55))	0.411
Mg		1.83 ± 0.42 (1.80 (1.60–2.00))	1.96 ± 0.29 (1.90 (1.80–2.10))	0.195
P		3.58 ± 1.31 (2.90 (2.60–4.60))	3.05 ± 0.82 (3.00 (2.50–3.10))	0.174
Max AST		36.24 ± 22.11 (33.00 (19.00–42.00))	62.71 ± 70.46 (38.00 (24.00–67.50))	0.296
Max ALT		28.65 ± 15.37 (28.00 (17.00–33.00))	50.45 ± 61.5 (50.45 (45.80–71.00))	0.272
Max ALKP		191.25 ± 85.08 (157.50 (133.00–242.50))	294.96 ± 300.93 (234.50 (159. -278))	0.189
Total Bill		1.18 ± 1.24 (0.70 (0.60–1.20))	2.83 ± 4.92 (0.70 (0.50–2.90))	0.825
Max PT		12.50 ± 0.84 (12.00 (12.00–13.00))	14.94 ± 8.20 (12.00 (12.00–12.50))	0.481
Max PTT		42.20 ± 17.66 (33.00 (28.00–50.00))	32.36 ± 11.34 (31.00 (28.00–37.00))	0.089
Max INR		1.69 ± 1.88 (1.00 (1.00–1.27))	4.67 ± 10.55 (1.00 (1.00–1.39))	0.639

*For quantitative variables: Mean ± SD(MED(p25-p75))

Mean and median of maximum creatinine during the hospitalization period was 2.59 ± 1.70 (IQR: 1.30–3.65) in RTRs and 1.10 ± 0.46 (IQR: 0.90–1.20) in the control group, compared with the baseline.

In terms of renal function, RTRs had a higher elevation in creatinine level than their non-RTR counterparts. A total of 13 out of 24 (54.1%) RTRs developed AKI, while 10 out of 40 (25%) patients experienced a similar situation in the control group (*P* = 0.001).

A lower mean and median level of absolute lymphocytic count (ALC) was noted in RTRs (820.93 ± 432.54) compared with non-RTRs (1794.89 ± 1376.89) (*P* = 0.002).

RTRs developed a lower platelet count as compared with their non-RTR counterparts; the mean platelet count in RTRs was 165.13 ± 81.95 versus 269.10 ± 141.25 in the control group (*P* = 0.002).

Other laboratory values anywhere not significantly different between the two groups (*P* > 0.05).

### Outcomes

Length of hospital stay and other outcomes are described in Table 4.

**Table 4:** Outcomes inRTRs and nonRTRs

		Group		*P*
		Transplant Pts	Non-Transplant Pts	
		Count (N %)	Count (N %)	
Duration of hospitalization		9.83 ± 7.29, 7.50(4.00–15.00)	8.62 ± 6.63, 6.00(4.00–11.00)	0.556
Discharge status	Discharge	18 (75.0)	35 (87.5)	0.173
	Expired	6 (25.0)	5 (12.5)	
Severity	Mild	0 (0.0)	0 (0.0)	0.524
	Moderate	7 (29.2)	10 (25.0)	
	Sever	8 (33.3)	11 (47.5)	
	Critical	9 (37.5)	19 (27.5)	
ICU admission	No	15 (65.2)	27 (73.0)	0.524
	Yes	8 (34.8)	10 (27.0)	
Ventilation type	No ventilation	0 (0.0)	5 (13.2)	0.068
	Nasal o2 or mask	14 (63.6)	23 (60.5)	
	NIV	2 (9.1)	6 (15.8)	
	MV	6 (27.3)	4 (10.5)	
O2satatadmission		90.74 ± 8.47(93.00(88.00–96.00))	89.26 ± 6.32(90.00(85.00–95.00))	0.148
RR at admission		19.70 ± 3.57(20.00(18.00–20.00))	19.49 ± 2.42(19.00(18.00–20.00))	0.940

Mean ± SD(MED(p25-p75))

The median age for KTRs who eventually expired post-transplantation was 11.5 years (IQR: 3–18).

There was no difference in the mean and median of duration of hospital stay between the two groups (9.83 versus 7.50 days, *P* = 0.556).

In-hospital mortality and ICU care requirements were not significantly different between the two groups (*P* = 0.173 and 0.524, respectively).

A lower proportion of RTRs (33.3%) manifested a severe course of infection than non-RTRs (47.5%). Conversely, more RTRs (37.5%) showed a critical status than their counterparts (27.5%); however, the difference was not statistically significant (*P* = 0.524).

A total of 6 (27.3%) KTRs and 4 (10.5%) non-KTRs underwent intubation and invasive MV; however, there was no statistically significant difference in types of ventilation administration between the two groups (*P* = 0.068) (Table 4).

ICU admission was associated with a higher mortality rate in RTRs (*P* = 0.007) compared to non-RTRs; however, no statistically significant difference was found in mortality rate between patients who were admitted to ICU and who were not (*P* = 0.172).

Receiving MV was associated with significant mortality in both populations (*P* < 0.001 and *P* = 0.002 in RTRs and non-RTRs, respectively).

No significant difference was found in the mean and median of admission O_2_ saturation between the patients (in both groups) who eventually expired and who did not (*P* = 0.796 versus *P* = 0.293, respectively).

In RTRs, a higher respiratory rate was responsible for higher mortality (*P* = 0.017) compared to the control group, in which respiratory rate was not significantly different between the deceased and survived patients (*P* = 0.471).

A lower proportion of statin users developed a critical status in the control group (1 [7.7%] versus 10 [40%] in statin and non-statin users, respectively) (P = 0.039). This difference was not significant among RTRs (P = 0.514) (Table 5).

**Table 5:** Correlation between underlying disease and drugs with outcomes

		Non-transplanted							
		EXPIRED	*P*	ICU admission	*P*	MV	*P*	Total adverse outcomes	*P*
PMH.HTN	No	0 (0.0)	0.022	6 (26.1)	0.580	0 (0.0)	0.015	6 (26.1)	0.340
	Yes	4 (25.0)		4 (28.6)		4 (28.6)		6 (37.5)	
PMH. diabetes	No	1 (3.7)	0.078	9 (33.3)	0.159	2 (7.7)	0.341	9 (33.3)	0.450
	Yes	3 (25.0)		1 (10.0)		2 (18.)		3 (25.0)	
PMH.CVA	No	4 (10.5)	0.897	10 (27.8)	0.730	4 (11.1)	0.892	12 (31.6)	0.692
	Yes	0 (0.0)		0 (0.0)		0 (0.0)		0 (0.0)	
Corticosteroid	No	5 (13.9)	0.573	8 (24.2)	0.291	3 (8.8)	0.372	11 (30.6)	0.392
	Yes	0		2 (50.0)		1 (25.0)		2 (50.0)	
		(0.0)							
ARB or ACEi	No	0 (0.0)	0.034	7 (28.0)	0.429	1 (4.2)	0.253	7 (28.0)	0.571
	Yes	3 (23.1)		2 (18.2)		2 (16.7)		4 (30.8)	
Atorvastatin	No	3 (12.0)	0.273	8 (34.8)	0.095	2 (8.7)	0.402	10 (40.0)	0.039
	Yes	0 (0.0)		1 (8.3)		0 (0.0)		1 (7.7)	
Warfarin	No	2 (5.6)	0.154	7 (21.2)	0.061	1 (2.9)	0.110	9 (25.0)	0.078
	Yes	1 (50.0)		2 (100.0)		1 (50.0)		2 (100.0)	

		t-Transplanted								
		Expired (%)	*P*	ICU admission (%)	*P*	MV (%)	*P*	Bad outcome (%)	Total (%)	*P*
Donor	Live	3	0.701	3	0.670	3	0.635	4	13	0.5677
		23.1		25.0		27.3		30.8	100.0	
	Deceased	1		1		1		1	5	
		20.0		20.0		20.0		20.0	100.0	
Duration of transplantation	Mean	11.17	0.210	10.00	0.373	11.17	0.165	10.33	8.96	0.294
	SD	7.25		6.46		7.25		6.12	4.94	
	Median	11.50		9.50		11.50		10.00	9.00	
	Percentile 25	3.00		4.00		3.00		5.00	5.00	
	Percentile 75	18.00		15.00		18.00		13.00	12.00	
PMH.HTN	No	0	0.384	1	0.709	0	0.342	1	3	0.668
		0.0		33.3		0.0		33.3	100.0	
	Yes	6		7		6		8	20	
		30.0		36.8		33.3		40.0	100.0	
PMH. diabetes	No	3	0.639	4	0.670	3	0.633	5	12	0.567
		25.0		36.4		27.3		41.7	100.0	
	YES	3		4		3		4	11	
		27.3		36.4		30.0		36.4	100.0	
PMH.CVA	NO	6	0.739	8	0.636	6	0.714	9	22	0.609
		27.3		38.1		30.0		40.9	100.0	
	Yes	0		0		0		0	1	
		0.0		0.0		0.0		0.0	100.0	
Cyclosporine	No	2	0.463	3	0.454	2	0.477	3	10	0.363
		20.0		30.0		22.2		30.0	100.0	
	Yes	4		5		4		6	13	
		30.8		41.7		33.3		46.2	100.0	
Tacrolimus	No	3	0.683	4	0.681	3	0.704	5	13	0.584
		23.1		33.3		25.0		38.5	100.0	
	Yes	2		3		2		3	9	
		22.2		33.3		25.0		33.3	100.0	
Sirolimus	No	6	0.739	8	0.636	6	0.714	9	22	0.609
		27.3		38.1		30.0		40.9	100.0	
	Yes	0		0		0		0	1	
		0.0		0.0		0.0		0.0	100.0	
Corticosteroid	No	0	0.750	0	_	0	_	0	1	0.625
		0.0		0.0		0.0		0.0	100.0	
	Yes	6		8		6		9	23	
		26.1		34.8		27.3		39.1	100.0	
ARB or ACEi	No	4	0.480	5	0.165	4	0.480	6	18	0.397
		22.2		27.8		22.2		33.3	100.0	
	Yes	2		3		2		3	6	
		33.3		50.0		33.3		50.0	100.0	
Atorvastatin	No	0	0.228	1	0.593	0	0.288	1	4	0.514
		0.0		25.0		0.0		25.0	100.0	
	Yes	6		7		6		8	20	
		30.0		35.0		30.0		40.0	100.0	
Warfarin	No	6	0.739	8	0.636	6	0.714	9	22	0.609
		27.3		38.1		30.0		40.9	100.0	
	Yes	0		0		0		0	1	
		0.0		0.0		0.0		0.0	100.0	

In the control group, angiotensin-​converting enzyme (ACE)/ Angiotensin Receptor Blocker (ARB) use, HTN, and receiving MV were factors significantly associated with in-hospital mortality, and statin use was significantly related to a lower rate of adverse outcomes (death, MV, and ICU care) (Table 5).

## Discussion

### General

The present study was designed with the intention of comparing RTRs and non-RTRs infected with COVID-19, particularly focusing on clinical symptoms, imaging features, disease severity, and outcomes between these populations, at the onset of the outbreak.

### Comorbidity

HTN was significantly higher in RTRs compared to the control group. The Benoteman et al. cohort study revealed that the most common comorbidity among RTRs infected with COVID-19 was HTN, and a study from Turkey found HTN and diabetes as the most common comorbidities among patients with kidney disease [13, 21]. These findings were expected due to the underlying disease, routine use of drugs such as steroids and CNIs, and lower glomerular filtration rate (GFR).

### Clinical symptoms

In our study, fever and cough were found to be the most common clinical presentations in RTRs, consistent with reported symptoms in immunocompromised patients in a recent systematic review [22]. However, in some other studies, fever was a less common symptom among RTRs [8].

According to a recent study, solid organ transplant patients presented more with dyspnea than the general population; conversely, in our study, dyspnea was significantly lower in RTRs than in the control group [22]. Cytokine release syndrome (CRS), which has been suggested as a damaging mechanism in COVID-19, can induce lung inflammation. Therefore, preventing or reducing the release of cytokines will reduce organ damage [23]. Thus, theoretically, the lower incidence of shortness of breath in RTRs might be due to the anti-inflammatory effect of maintenance anti-rejection therapy in these patients.

A recent study reported diarrhea as a common clinical presentation of SARS-CoV-2 infection in RTRs, in which the prevalence was remarkably higher than that expressed in the population without kidney disease [24]. It is speculated that gastrointestinal (GI) symptoms are exacerbated by immunosuppressive agents and thus are probably more frequent in RTRs infected with COVID-19 than the general population [24]; however, we have not found any significant difference in GI symptoms between the two study groups.

### Immunosuppression

RTRs may be more susceptible to infection due to their immunosuppression and burden of comorbidities, including diabetes, HTN, and cardiovascular disease. Although the definitive effect of immunosuppression on host immune response is unknown yet, it has been speculated that chronic immunosuppression may play a role as a protector against hyper-inflammatory response and cytokine storm severity in RTRs with COVID-19 [25]; thus the possibility of subsequent respiratory damage resulting from elevated cytokines would be mitigated. In view of this, it is assumed that infection with COVID-19 might not result in worse consequences in patients under immunosuppression agents chronically. Additionally, the protective role of chronic use of CNIs has been suggested in COVID-19 infected patients [26]**.**

On the other hand, being on chronic immunosuppression, especially at the first phase of infection, has been thought to increase morbidity and mortality owning to the altered immune system during the early episode of SARS-CoV-2 infection, during which a strong response is required in order to overcome viral replication and disease progression; moreover, immunosuppression puts individuals at higher risk of secondary infections [25, 27].

In our study, Mycophenolate mofetil (MMF) was discontinued in all patients at the time of hospitalization, and CNIs were either reduced or withdrawn based on the severity of illness, with the intention of minimizing the adverse effects of CNIs and antimetabolites on the clinical course of viral pneumonia [25].

There have been arguments that immunosuppressive (IS) agents’ reduction or withdrawal predisposes individuals with hyperinflammatory response to allograft rejection [6]; however, in our study, despite a remarkable dose reduction of IS medications through the admission period, none of the KTRs experienced allograft rejection, because none of them developed progressive kidney failure and renal replacement therapy requirements and gradually recovered without antirejection therapy. Lack of rejection might have been modulated by the concomitant rise of corticosteroids to stress dose in the setting of reducing or stopping CNIs and antimetabolites. The protective role of anti-inflammatory drugs against rejection has also been suggested in patients in whom CNIs were stopped as a result of severe infection [3].

Importantly, immunosuppression management should be considered in each KTR infected with COVID-19 on a case-by-case basis.

### Mortality and ICU admission

Based on our results, adverse outcomes, including in-hospital mortality, ICU admission, and MV requirements, were not significantly different between RTRs and non-RTRs.

Although the rate of ICU admission was not significantly different between the groups, it was associated with higher mortality in RTRs. RTRs generally have greater predisposition to bacterial super infection due to the impaired immune system as a result of antirejection regimes. Concurrent superinfection might have been responsible for higher mortality among ICU-admitted patients in this group.

In this study, the mortality rate in RTRs (25%) was two times higher than that of the control group (12.5%); it was not statistically significant, however. The higher percentage of mortality in RTRs could be attributed to not only higher burden of comorbidities, single functioning kidneys, and worse laboratory parameters in these patients, but also the inefficient immune function at the early phase of the infection, at which a strong immune response is required to subside the viral replication and overload.

Meanwhile, the non-significant difference in mortality between the two population groups, investigated in this study, might be due to the immune balancing and anti-inflammatory effect of immunosuppressive agents used by RTRs. The chronic use of immunosuppressive drugs might modulate cytokine release storm, which could be potentially responsible for a higher rate of mortality during the late phase of COVID-19 infection. Therefore, a lower than expected mortality rate in RTRs and thus a non-significant difference in mortality between RTRs and non-RTRs, might have been engendered by the mitigated cytokine release storm (CRS), which is presumably the result of chronic immunosuppression in RTRs. (Reviewer 2- comment 1).

Similar to our study, some previous observational studies had noted a non-significant difference in mortality and adverse outcomes (death or ICU admission) between RTRs and non-RTRs [13, 14].

Our study is also in line with a study conducted in the United States, in which no significant difference in mortality, risk of MV, and hospital stay duration was detected between patients who were on chronic immunosuppression and immune-competent patients hospitalized due to COVID-19 infection [27], and another study revealing a similar risk of mortality, ARDS, and organ support between solid organ transplant (SOT) recipients and a control group [10]**.**

In terms of COVID-19 infection severity, no significant difference was detected between the two groups, whereas, in a recently conducted case control study, severe to critical situations were more detected in patients with kidney disease compared to patients without underlying kidney disease [13]. (Reviewer 2-comment 9).

### Antivirals

In our study, hydroxychloroquine (HCQ) and lopinavir/ritonavir were administered to the majority of the study population. Later, CNIs were withdrawn in all patients undergoing Kaletra treatment. Yet, quite a good number of these patients showed adverse events; moreover, there was no evidence of favorable outcomes neither in RTRs nor in the control group receiving these drugs. This finding is in line with a meta-analysis that did not find a lower risk of mortality, MV, and hospital stay duration in confirmed-COVID-19 patients treated with HCQ and Kaletra and a randomized trial in which no benefits of treatment with HCQ and Kaletra were found in the clinical course of patients with COVID-19 [28, 29].

### Laboratory data

In our study, a higher percentage of RTRs developed AKI than the control group. A recent investigation also showed a significantly higher incidence of AKI in RTRs than the general population [14]. RTRs are vulnerable to contributing factors of AKI etiologies during the COVID-19 infection course, such as acute rejection, hemodynamic imbalance, volume depletion, drug toxicity, and high fever [30]. Thus, prerenal azotemia, acute tubular necrosis, or other possible etiologies of AKI may get complicated more in RTRs than the general population. The reason may be the single functional kidney, lower tolerance to drugs, and potential immunological damage [15]. In this case, a renal biopsy is needed to determine the definitive cause of AKI. (Reviewer 2-comment 5).

Regarding the role of renin-angiotensin system (RAS) inhibitors in the outcome of COVID-19 infected non-RTRs, we have found that ACE/ARB treatment history is associated with mortality in this group. Contrary to our finding, a recent systematic review confirmed that prior ACE/ARB treatment is not responsible either for higher morality or for disease severity in the general population [31].

Patients on RAS inhibitors were mostly those with confirmed comorbidities such as HTN that could be potentially responsible for poor outcomes among ACE/ARB users.

In terms of statins, statin users developed a less critical situation than non-statin users in non-RTRs. This finding was expected because statins are known to reduce the possibility of CRS via an anti-inflammatory and immune modulatory effect, in line with a recent study in which statin users required less ICU admission [32]. Improved outcomes only in our control group reflect that anti-inflammatory effects of statins may be more prominent in the absence of chronic use of immunosuppressive agents as in non-RTRs.

Lymphopenia and thrombocytopenia as indicating factors of mild inflammation were detected notably more in RTRs than in non-RTRs. In agreement with our results, a recent case–control study reported a significantly lower frequency of thrombocytopenia and lymphopenia in patients without any underlying kidney disease than RTRs, and Chronic kidney disease (CKD) and end-stage renal disease (ESRD) patients [13]. Besides, in another study, lymphocytopenia was a frequent finding among immunocompromised organ transplant recipients with COVID-19 [33]. Lower lymphocyte and platelet counts in RTRs may be a result of routine immunosuppressive agent use like antimetabolites and chronic disease [34].

In our study, 52% of RTRs were found to have higher levels of CRP compared with the control group. In line with a study conducted in Turkey, CRP increase was higher (36.7%) in KTRs compared with their control group [13].

In terms of CT scan features, 95% of patients in each group had GGO, as a highly suggestive pattern of COVID-19 pneumonia, followed by consolidation as another prevalent lesion mostly accompanied by other lesion types, with no significant difference between them.

Crazy-paving, linear opacity, bronchiectasis, and consolidation alone as findings associated with severe to critical clinical status were not significantly different between the two groups and were roughly in the same range as in previous studies on the general population [35, 36].

Pleural effusion (23%), as an extrapulmonary lesion indicator of severe inflammation and viral load [35], was frequently more among critical and severe patients, according to previous studies. Surprisingly, in our study, it was significantly higher in the control group and none of the RTRs had this feature. Lack of this finding among RTRs might be due to the protective role of immunosuppressive agents against cytokine release, which might be responsible for pleural effusion.

Regarding the distribution of lesions, a high proportion of lesions were peripherally and bilaterally distributed and were multifocal, again with no difference between the two groups and the proportions were consistent with previous studies [35, 36]. The total CT score and number of involved lobes were almost the same, indicating that the severity and extent of COVID-19 pneumonia were not different between the two groups.

## Limitations

Our study has certain limitations. First, our sample size was small and follow-up duration was short; thus, complementary investigations with larger sample sizes and longer follow-up duration are recommended for drawing firm conclusions.

Second, it lacked relevant data due to the scarcity of registry databases at the beginning of the pandemic in our center.

SARS-CoV-2 RT-PCR test was challenging due to inadequate supply of COVID-19 diagnostic test kits at that time. Hence, COVID-19 was confirmed in only 26.5% of patients with positive RT-PCR, and the remaining patients in whom RT-PCR was negative or did not have a confirmation test were included if lung HRCT lesions and clinical symptoms were highly suspicious of SARS-CoV-2 infection.

We did not measure some inflammatory biomarkers, e.g. IL-6 and D-dimer, and also quantitative CRP, which could be potential predictors of inflammation and thrombotic events as well as disease severity.

Although there was an 8-month follow-up of RTRs, for the control group, since their initiation of clinical symptoms, there was just a 2-month follow-up. However, no major complications were observed over the follow-up period neither in RTRs nor in the control group.

## Conclusion

In this case–control study of RTRs and non-RTRs, no significant difference was found in the severity, patterns of pulmonary involvement, risk of MV, ICU admission, length of hospital stay, and mortality between KTRs under chronic immunosuppression and the general population. Further investigations with larger sample sizes are recommended for reassurance and firm conclusions.

## Data Availability

The data supporting the findings of this study are available from the corresponding author upon reasonable request.

## Abbreviations

ACE: Angiotensin-converting enzyme
AKI: Acute kidney injury
ALC: Absolute lymphocytic count
ARB: Angiotensin receptor blocker
ARDS: Acute respiratory distress syndrome
CKD: Chronic kidney disease
CNI: Chronic renal failure
COPD: Chronic obstructive pulmonary disease
CRP: C-reactive protein
CRS: Cytokine release syndrome
CVA: Cerebrovascular accident
DVT: Deep venous thrombosis
ESR: Erythrocyte sedimentation rate
ESRD: End-stage renal disease
GFR: Glomerular filtration rate
GGO: Ground-glass opacity
GI: Gastrointestinal
HCQ: Hydroxychloroquine
HF: Heart failure
HRCT: High-resolution computed tomography
HTN: Hypertension
ICU: Intensive care unit
IHD: Ischemic heart disease
IQR: Interquartile range
IS: Immunosuppressive
IVIG: Intravenous immunoglobulin
KDIGO: Kidney Disease Improving Outcomes
KTR: Kidney transplant recipient
MMF: Mycophenolate mofetil
MV: Mechanical ventilation
NIV: Non-invasive ventilation (Reviewer 2- comment 11)
PHEIC: Public Health Emergency of International Concern
PTE: Pulmonary thromboembolism
RAS: Renin-angiotensin system
RT-PCR: Reverse transcription-polymerase chain reaction
RTR: Renal transplant recipient
SOT: Solid organ transplant
